# Exploring Medical Doctors’ Confidence in Artificial Intelligence: The Role of Specialty, Experience, and Perceived Job Security

**DOI:** 10.3390/healthcare13182377

**Published:** 2025-09-22

**Authors:** Fahad Abdulaziz Alrashed, Tauseef Ahmad, Ahmad Othman Alsabih, Shimaa Mahmoud, Muneera M. Almurdi, Hamza Mohammad Abdulghani

**Affiliations:** 1 Department of Medical Education, College of Medicine, King Saud University, Riyadh, P.O. Box 2925, Riyadh 11461, Saudi Arabia; tahmad@ksu.edu.sa; 2 Department of Physiology, College of Medicine, King Saud University, Riyadh, P.O. Box 2925, Riyadh 11461, Saudi Arabia; aalsabih@ksu.edu.sa; 3 Anatomy Department, Stem Cell Unit, College of Medicine, King Saud University, Riyadh, P.O. Box 2925, Riyadh 11461, Saudi Arabia; mshimaa@ksu.edu.sa; 4 Department of Health Rehabilitation Sciences, College of Applied Medical Sciences, King Saud University, Riyadh, P.O. Box 10219, Riyadh 11433, Saudi Arabia; malmurdi@ksu.edu.sa; 5 Department of Medical Education and Family Medicine, College of Medicine, King Saud University, Riyadh, P.O. Box 2925, Riyadh 11461, Saudi Arabia; hamzaabg@ksu.edu.sa

**Keywords:** artificial intelligence, medical doctors, confidence, specialty, experience, healthcare, AI adoption

## Abstract

**Background:** Artificial intelligence (AI) is increasingly integrated into healthcare, offering transformative potential across diagnostics, treatment, and clinical decision-making. As its adoption grows, understanding how medical doctors perceive and respond to AI, particularly in relation to their specialty, experience, and job security, is critical for effective implementation and acceptance. This study investigates the confidence of medical doctors in AI technologies and their role in healthcare, focusing on the impact of specialty, experience, and perceived job security. **Method:** A cross-sectional survey was conducted among 187 medical doctors across various specialties in Riyadh, Saudi Arabia, with a final sample of 176 participants. The survey assessed awareness, confidence, and concerns regarding AI integration into clinical practice. The survey was conducted across multiple healthcare hospitals in Riyadh, Saudi Arabia. Hospitals from both public and private sectors were included to ensure a diverse sample of healthcare professionals from different organizational structures. **Results:** A statistically significant association was found between specialty and confidence level (χ^2^ = 14.5, *p* = 0.001). Among specialists, the majority (80%) reported high confidence in AI use compared to 45% of general practitioners and 38% of surgeons. Conversely, moderate confidence was most common among surgeons (46%), followed by general practitioners (35%) and specialists (13%). Additionally, participants with 11–20 years of experience reported the highest confidence, whereas those aged 55+ years showed the lowest perceived impact of AI on patient outcomes. Multivariate regression analysis identified specialty as the strongest predictor of AI confidence, with specialists being four times more likely to express high confidence in AI use (β = 0.89, *p* = 0.001) compared to general practitioners. Job displacement concerns negatively influenced confidence in AI, while age and years of experience had less impactful effects. **Conclusions:** The study concludes that addressing barriers to AI adoption will be crucial for enhancing its integration into healthcare and improving patient care. These findings underscore the importance of specialty-specific training and highlight the need for targeted educational programs, particularly for lower confidence groups such as general practitioners and surgeons. Lower confidence levels in these groups may result in a hesitant or incorrect use of AI tools, potentially compromising patient safety. Therefore, equipping all healthcare professionals with the necessary knowledge and confidence is essential for the safe and effective use of AI in clinical practice.

## 1. Introduction

Artificial intelligence (AI) has emerged as a transformative force in various industries, with healthcare being one of the primary sectors benefiting from its applications. AI technologies are increasingly being integrated into medical practices, from diagnostic support and predictive analytics to patient management and personalized treatment plans [1,2,3]. This growing presence of AI in the medical field raises important questions about how medical professionals perceive these advancements and their readiness to embrace AI-driven solutions. Despite the potential for AI to revolutionize patient care and improve health outcomes, the perspectives of medical doctors regarding AI adoption, its challenges, and its implications for clinical practice remain a vital area of exploration.

Recent studies highlight that AI in healthcare is viewed as a tool to augment the capabilities of healthcare providers rather than replace them [4,5,6]. AI-based diagnostic systems, such as those using deep learning to analyze medical imaging, have shown promise in detecting conditions like cancer and cardiovascular diseases with high accuracy [7,8,9]. These innovations aim to reduce human error and enhance diagnostic speed, which is crucial in fast-paced medical environments. However, concerns remain about the interpretability of AI systems, as well as their ability to make context-aware decisions that align with the nuanced understanding of human clinicians [7,10]. Despite these potential benefits, the integration of AI into healthcare is not without challenges. A significant barrier to adoption is the skepticism among healthcare professionals regarding the reliability and ethical implications of AI technologies [11]. Medical doctors, who are trained to make complex, often subjective decisions based on a combination of medical knowledge and patient context, may be hesitant to fully trust AI systems in clinical practice [12]. Furthermore, there is a gap in understanding the implications of AI on physician–patient relationships, with concerns about dehumanization and the loss of empathetic care [13,14]. Another critical factor is the need for proper training in AI technologies. While the integration of AI into medical curricula is slowly increasing, many healthcare professionals have expressed concerns over their ability to work alongside AI without adequate preparation [13]. Additionally, the potential for AI to exacerbate existing healthcare disparities if algorithms are trained on biased data raises concerns about equity in healthcare delivery [15,16]. With the potential to enhance diagnostic accuracy, optimize patient care, and reduce operational inefficiencies, AI has become an essential tool in modern medicine. A global survey involving clinicians across 20 countries found that more than two-thirds (68%) were optimistic about AI’s potential to enhance diagnostic accuracy and efficiency, yet only 31% had actually used such tools in clinical practice during the previous year. Major barriers to adoption included a lack of training (62%), doubts regarding efficacy (48%), and an insufficient clinical validation of AI applications (45%) [17]. Earlier research in Korea echoed similar trends: physicians recognized AI’s utility in diagnosis and treatment planning, but only a minority believed AI would surpass human clinicians diagnostically. Importantly, more experienced physicians expressed lower trust in AI’s superiority compared to medical students or early-career practitioners [18,19]. A focused study in Jeddah, Saudi Arabia, revealed that while 76% of physicians agreed on the accuracy of AI systems and 60% acknowledged their efficiency, actual usage was low, with only 25.9% reporting use in the past year. These findings highlight a gap between confidence in AI capabilities and its actual deployment in practice [20]. However, implementation remains a topic of considerable debate among medical professionals. While AI promises numerous benefits, questions regarding trust, ethical considerations, and its role in the physician–patient relationship persist [21,22]. In Saudi Arabia, research on physicians’ perspectives toward AI integration is limited, especially regarding its role in diagnostics, decision-making, and patient outcomes. This lack of context-specific evidence highlights the need to explore how medical doctors in Saudi Arabia perceive AI, which is essential to identify both challenges and opportunities for its effective adoption. These challenges require ongoing dialog within the medical community to ensure AI is used ethically and effectively. The primary aim of this study is to assess medical doctors’ confidence levels in integrating artificial intelligence (AI) into clinical practice. The secondary aims are to examine how this confidence is influenced by key factors such as medical specialty, years of professional experience, and perceived job security. Additionally, the study seeks to explore doctors’ views on the potential impact of AI on diagnostics, decision-making, and patient outcomes.

## 2. Methods

A cross-sectional survey was conducted to gather data on the perspectives of medical doctors regarding the use of artificial intelligence (AI) in healthcare. The study aimed to assess the level of confidence and concerns about AI technologies in clinical practice among physicians across a range of medical specialties. By utilizing a survey approach, this study was able to capture a broad range of opinions from medical doctors practicing in different settings, thereby enhancing the generalizability of the findings to a wide population of healthcare professionals.

### 2.1. Study Setting

The survey was conducted across multiple healthcare hospitals in Riyadh, Saudi Arabia. Hospitals from both public and private sectors were included to ensure a diverse sample of healthcare professionals from different organizational structures. The selected hospitals varied in size and the range of services offered, from large tertiary care centers to smaller clinics. This diversity in hospital types and locations allowed for the exploration of how different practice environments influence medical doctors’ attitudes toward AI technologies.

### 2.2. Survey Instrument

Data collection was carried out using an online self-administered questionnaire, which was developed following an extensive literature review to align with the objectives of the study. The questionnaire was divided into several key sections such as demographics, awareness of AI, confidence in AI, and ability to assist with diagnostics to evaluate different aspects of AI’s role in healthcare. The initial version of the questionnaire contained thirteen items which were subjected to thorough discussion by a panel of four consulting medical doctors (one orthopedic surgeon, two family medicine doctors, and one general surgeon). After two meetings, consensus was reached on nine items to be included. Both the consulting team and the ethics committee recommended conducting a pilot study before proceeding with the main study. Accordingly, a pilot study was conducted with 11 participants, focusing on the use of AI in healthcare. The internal consistency of the instrument was assessed using Cronbach’s alpha, yielding a reliability score of 0.81 for the nine finalized items. The first section included three questions related to the demographic information of the participants. The second section consisted of three items assessing doctors’ confidence levels and perceptions of job displacement, measured using a three-point Likert scale with the following response options: (3 = “High confidence”, 2 = “Moderate confidence”, and 1 = “Low confidence”). The survey was distributed electronically via email to the participating doctors, and responses were collected over a period of six weeks. The doctors were informed of the purpose of the study and assured that their responses would be kept confidential. Consent was obtained electronically prior to participation, and all respondents were given the opportunity to withdraw from the study at any time without consequence. In addition to the electronic survey, follow-up reminders were sent to non-respondents to ensure a high response rate. The completion time for the survey ranged from 7 to 10 min.

### 2.3. Study Participants

For the purposes of analysis, physicians were classified into three main categories, namely general practitioners (GPs), surgeons, and medical specialists. This classification was determined after discussion within the research team, using the participants’ professional roles and hospital appointments to ensure accuracy. Prior to completing the survey, all physicians were informed about the objectives of the study, and written informed consent was obtained. Survey links were then distributed via email or WhatsApp only to those who agreed to participate. The survey was distributed to 187 medical doctors across various specialties, including general practitioners, specialists (e.g., cardiologists, neurologists, and oncologists), and surgeons. The inclusion criteria were licensed medical doctors with at least five years of clinical experience and doctors who were actively involved in patient care at the time of the survey. The sample size was chosen to ensure a diverse representation of healthcare professionals, reflecting a range of specialties, practice environments, and experience levels. This approach allowed for the examination of differences in perceptions based on specialty, geographical location, and years of experience.

### 2.4. Sample Size

Sample size= Z_1−a/2_^2^ (SD)^2^/d^2^

Here Z_1−a/2_^2^ = standard normal variate (at 5% type 1 error (*p* < 0.05), it is 1.96 and at 1% type 1 error (*p* > 0.01), it is 2.58). In our case, *p* values are considered significant below 0.05; hence, 1.96 is used in the formula.

d = Absolute error 5%

SD = expected proportion in population based on current studies, previous studies, or pilot studies.

(In many previously published studies, such as Physicians’ attitudes and knowledge toward artificial intelligence, the sample size was 114 [23]; some studies reported 74 out of 142 (52.12%) [24], 89 out of 164 (54.3%) [25], and 107 out of 301 (35.6%) [26].)

Sample size = 1.96^2^ × (35.6)^2^/5^2^ = 197.8

The sample size, if 35.6% according to previous published studies, is 198.

The minimum required sample size for conducting such studies has been estimated to be approximately 198 participants based on previously published research. However, since our study was limited to physicians in a single city (Riyadh), we aimed to achieve a comparable number of participants, as close as possible to this requirement.

### 2.5. Data Analysis

Descriptive statistics were employed to summarize demographic data, such as age, specialty, and years of experience. Chi-squared tests were used to determine any statistically significant associations between medical specialties and confidence levels in AI’s diagnostic applications. The association with each healthcare and medical specialty was determined using odds ratios (ORs) along with 95% confidence intervals (CIs). A *p* value of <0.05 was used to report the statistical significance of the results. Data were analyzed using statistical package for social sciences program 24.

## 3. Results

In the current study, a total of 187 questionnaires were distributed to medical doctors. Of these, 182 responses were received. After careful review, six responses were excluded due to incomplete data, resulting in a final sample size of 176, yielding a response rate of 94.1%. Among them, the majority of participants were between 35 and 44 years old (38.6%), followed by those aged 25–34 years (31.8%), 45–54 years (23.9%), and 55 years or older (5.7%). Regarding medical specialties, most respondents were specialists (51.1%), followed by general practitioners (34.1%) and surgeons (14.8%). In terms of professional experience, 40.9% of participants had 5–10 years of experience, 39.8% had 11–20 years, and 19.3% had more than 21 years of experience (Table 1).

Table 2 summarizes the association between medical specialty and self-reported confidence in using artificial intelligence (AI). A statistically significant association was found between specialty and confidence level (χ^2^ = 14.5, *p* = 0.001). Among specialists, the majority (80%) reported high confidence in AI use compared to 45% of general practitioners and 38% of surgeons. Conversely, moderate confidence was most common among surgeons (46%), followed by general practitioners (35%) and specialists (13%). Low confidence levels were reported by 20% of general practitioners, 15% of surgeons, and only 7% of specialists. These findings suggest that specialists tend to have greater confidence in using AI compared to general practitioners and surgeons.

Table 3 illustrates participants’ perceptions of the impact of artificial intelligence (AI) on patient outcomes, analyzed by years of experience and age group using a five-point Likert scale. Participants with 11–20 years of experience reported the highest mean score (4.3), followed by those with 5–10 years (4.1). Participants with 21+ years of experience had the lowest mean score (3.8), suggesting a slightly lower perceived impact of AI on patient outcomes with increasing experience. Regarding age, the 25–34 age group reported the highest mean score (4.2), followed closely by the 35–44 group (4.1). Perceived impact decreased with age, with the 45–54 group scoring 3.9 and the 55+ group reporting the lowest score (3.5).

Table 4 shows the correlation between AI confidence levels and medical specialty, expressed in terms of odds ratios for high confidence. Specialists had the highest proportion of participants reporting high confidence in AI use (80%), with an odds ratio of 4.00, indicating they were four times more likely to report high confidence compared to low or moderate confidence. In contrast, general practitioners had a high confidence rate of 45%, with an odds ratio of 0.82, while surgeons reported the lowest high confidence rate at 38%, with an odds ratio of 0.63. Based on Table 4, we calculated the odds of being highly confident in AI for specialists (OR = 4) compared to general practitioners (OR = 0.82) and surgeons (OR = 0.63).

The OR of general practitioners (GPs) and specialists = (High Confidence of AI/Low and Moderate Confidence) of specialists/(High Confidence of AI/Low and Moderate Confidence) of GPs.

The OR of GPs and specialists = (72/18)/(27/33) = 4.00/0.82 = 4.88 OR.

The odds of a specialist being highly confident in AI are 4.88 times higher than those of a general practitioner.

The OR of surgeons and specialists = (High Confidence of AI/Low and Moderate Confidence) of specialists/(High Confidence of AI/Low and Moderate Confidence) of surgeons.

The OR of GPs and specialists = (72/18)**/**(10/16) = 4.00/0.63 = 6.35 OR.

The odds of a specialist being highly confident in AI are 6.35 times higher than those of a surgeon. This study found that specialists are significantly more likely to be confident in AI’s abilities, likely due to their higher exposure to AI-driven tools in fields such as radiology, oncology, and pathology. Table 5 shows a multivariate regression analysis to explore the factors that predict confidence in AI among doctors. The current results showed that specialty has the strongest effect on confidence in AI (β = 0.89, *p* = 0.001), indicating that doctors in specialized fields are significantly more likely to be confident in AI. The majority of doctors’ concerns are about job displacement due to AI exposure (β = −0.12, *p* = 0.003). Furthermore, age and years of experience both have a positive but weaker influence on confidence in AI, though age approaches significance (*p* = 0.058).

## 4. Discussion

The results of this study provide valuable insights into the self-reported confidence levels in the use of artificial intelligence (AI) across different medical specialties. A total of 176 respondents participated in the study, yielding a high response rate of 94.1%, which is indicative of strong engagement among the medical professionals surveyed. The majority of respondents were between 35 and 44 years old (38.6%), and the largest group by specialty was specialists (51.1%), followed by general practitioners (34.1%) and surgeons (14.8%). This distribution is representative of the varying roles and expertise within the healthcare sector, which is important when interpreting the results related to AI confidence in terms of medical specialty and confidence in using AI. Specifically, specialists reported the highest levels of confidence (80%), which aligns with previous studies suggesting that medical professionals with specialized training often have more exposure to advanced technologies, including AI, and thus may feel more competent in utilizing these tools; they have greater exposure to advanced technologies, particularly in diagnostic fields like radiology and cardiology, where AI tools are already in use [4,27,28,29,30]. On the other hand, general practitioners (45%) and surgeons (38%) reported lower levels of high confidence. This trend could be attributed to several factors, including a perceived lack of training in AI for general practitioners, who are often more focused on primary care and may have less access to cutting-edge AI tools. Surgeons, while specialized in their field, might also have limited exposure to AI, particularly in the clinical settings they operate in, where hands-on patient care is often emphasized over technological integration [1,31,32,33,34]. Furthermore, moderate confidence was most common among surgeons (46%), followed by general practitioners (35%) and specialists (13%). This finding suggests that while specialists may have high confidence in AI, the use of AI in surgical practice may still be seen as supplementary rather than integral, leading to a more cautious but moderate approach. General practitioners’ moderate confidence (35%) may reflect a gap in AI training and the perceived applicability of AI tools in primary care settings. Moreover, the lower confidence levels, particularly among general practitioners (20%) and surgeons (15%), highlight a potential barrier to the broader implementation of AI in healthcare. This low confidence could be linked to a lack of exposure to AI tools, concerns about the complexity of AI systems, or doubts about their effectiveness and reliability. Previous research has identified that confidence in new technologies is often influenced by training, experience, and perceived ease of use [27,35,36,37,38]. AI continues to play a larger role in healthcare, and targeted educational programs and hands-on training may be essential to bridge this confidence gap across specialties [39]. A study in Saudi Arabia found that healthcare providers’ knowledge and attitude toward AI were strong predictors of patient safety culture; those with limited understanding or less favorable attitudes had weaker safety culture scores [40]. In Saudi Arabia, a lack of AI training correlated with less knowledge, less positive attitudes, and weaker patient safety culture, meaning safety risk behaviors or misuse are more likely [40].

This study examines the impact of AI on patient outcomes in relation to doctors’ years of experience. The participants with 11–20 years of experience reported the highest mean score (4.3), suggesting they see AI as a valuable tool in clinical practice. This group likely balances sufficient clinical exposure with active engagement in ongoing professional development and technological adaptation. Moreover, those with 21+ years of experience reported a lower perception score (3.8), which may reflect a more cautious attitude toward newer technologies or less exposure during earlier stages of their training [41,42]. In the current study, age was found to influence perceptions. Doctors aged 25–34 reported the highest impact score (4.2), followed closely by those aged 35–44 (4.1). Confidence decreased with age, with the 55+ group reporting the lowest score (3.5) in this study. This trend may reflect limited formal education in AI or concerns about replacing clinical judgment with algorithm-driven decisions [43]. Furthermore, in the current study, a significant variation in confidence levels in artificial intelligence (AI) across medical specialties was reported. Specialists showed the highest proportion of high AI confidence (80%) compared to general practitioners (GPs) at 45% and surgeons at 38%. The odds of a specialist being highly confident in AI were 4.88 times higher than those of a GP and 6.35 times higher than those of a surgeon. These findings suggest a strong association between specialty type and AI confidence. This trend may reflect the nature of clinical exposure to AI. Specialists, particularly in fields such as radiology, oncology, and pathology, are more likely to work with AI-enhanced diagnostic systems and decision support tools [10]. Their familiarity with these applications likely fosters greater trust and perceived utility, enhancing confidence in AI integration. In contrast, general practitioners and surgeons may have fewer opportunities to interact with AI technologies such as clinical decision support systems or surgical robotics in their daily workflows, contributing to lower confidence levels [44,45]. Additionally, the current study showed that specialty emerged as a significant positive predictor (β = 0.89, *p* = 0.001), indicating that specialists are more likely to report higher confidence in AI. This aligns with prior studies suggesting that specialists, particularly in fields like radiology and oncology, are more frequently exposed to AI tools and thus develop greater trust and familiarity with their use in clinical practice [10,44,46]. Moreover, job displacement concerns had a statistically significant negative association with AI confidence (β = −0.12, *p* = 0.003), reflecting the apprehension among healthcare professionals about the potential of AI to replace human roles. This concern has been widely reported in the literature, where the fear of automation is linked to reduced acceptance of AI, particularly in roles perceived as cognitively or diagnostically replaceable [47]. The negative association between job displacement concerns and AI confidence suggests that physicians who fear automation are less likely to trust or adopt AI in practice. This reflects a broader psychological response observed across professions, where workers perceive new technologies as a threat to their professional identity and job security [48]. In healthcare, this fear is particularly pronounced in specialties heavily reliant on diagnostic tasks, such as radiology and pathology, which are seen as more vulnerable to AI replacement [18]. As a result, anxiety about losing professional autonomy or being replaced may hinder clinicians’ willingness to engage with AI tools, despite their potential benefits [49]. Furthermore, age and years of experience were not statistically significant predictors (*p* > 0.05), though age approached significance (*p* = 0.058). These findings suggest that while older or more experienced clinicians may be somewhat less confident in AI, these factors alone do not strongly predict confidence when accounting for other variables.

### Limitations

The present study provides valuable insights into the confidence of medical doctors in integrating artificial intelligence (AI) into clinical practice; however, several limitations should be acknowledged. First, the cross-sectional design restricts the ability to establish causal relationships between variables such as specialty, years of experience, and AI confidence. Second, the study relied on self-reported data, which is inherently subject to recall and social desirability biases, potentially influencing how participants rated their confidence levels. Third, the sample was geographically restricted to Riyadh, Saudi Arabia, which may limit the generalizability of the findings to other regions or healthcare systems with different levels of AI adoption and infrastructure. Additionally, the study focused exclusively on medical doctors and excluded other healthcare professionals such as nurses, pharmacists, and allied health practitioners, who increasingly interact with AI systems in multidisciplinary clinical environments. The survey also concentrated on a limited range of specialties without exploring fields where AI integration may be more advanced or emerging. Finally, the study did not specify the types of AI applications being assessed, which constrains the ability to differentiate confidence levels across diagnostic, predictive, or administrative technologies.

## 5. Conclusions

Overall, the study highlights a clear association between age, experience, and perceptions of AI’s role in patient care. Mid-career and younger physicians exhibit greater optimism about AI’s impact, indicating that future implementation strategies must be inclusive and adaptive to the varying comfort levels and expectations within the healthcare workforce. The current study also highlights significant differences in confidence levels regarding AI between medical specialties, with specialists demonstrating the highest confidence. These findings suggest that specialty-specific AI training programs are essential for promoting the effective use of AI across all areas of healthcare. Addressing the barriers to AI adoption, particularly for general practitioners and surgeons, will be crucial in ensuring that AI technologies can be fully integrated to enhance patient outcomes and improve healthcare delivery.

### Recommendations

Based on the findings, it is recommended that targeted AI education and training programs be developed for general practitioners and surgeons, who demonstrated lower levels of confidence in AI compared to specialists. Tailored interventions should address the specific challenges of these groups and include practical, hands-on components to build familiarity, trust, and competence in using AI technologies. From a translational perspective, integrating AI education into undergraduate medical curricula and continuing professional development programs can help bridge existing knowledge gaps across different specialties and levels of experience. At the policy level, these insights can inform national health strategies by prioritizing specialty-specific training initiatives to ensure the equitable adoption of AI across the healthcare system. Additionally, future research should investigate the longitudinal effects of AI exposure on confidence and explore contextual factors such as organizational culture, regulatory frameworks, and healthcare infrastructure, which may influence AI acceptance and utilization.

## Figures and Tables

**Table 1:** Demographic information of the study.

Items	Category	Number of Doctors (N = 176)
Age	25–34 years	56 (31.8%)
	35–44 years	68 (38.6%)
	45–54 years	42 (23.9%)
	55+ years	10 (5.7%)
Specialty	General Practitioners	60 (34.1%)
	Specialists	90 (51.1%)
	Surgeons	26 (14.8%)
Years of Experience	5–10 years	72 (40.9%)
	11–20 years	70 (39.8%)
	21+ years	34 (19.3%)

**Table 2:** Association between specialty and confidence in AI.

Confidence Level	General Practitioners (*n* = 60)	Specialists (*n* = 90)	Surgeons (*n* = 26)	χ^2^ (*p*)
High Confidence	27 (45%)	72 (80%)	10 (38%)	14.5 (0.001)
Moderate Confidence	21 (35%)	12 (13%)	12 (46%)	
Low Confidence	12 (20%)	6 (7%)	4 (15%)	

**Table 3:** Participants’ perception of AI and impact on patient outcomes based on years of experience and age group (mean score 1–5 scale).

Items	Categories	Mean Score Scale
Years of Experience	5–10 years	4.1
	11–20 years	4.3
	21+ years	3.8
Age group	25–34 years	4.2
	35–44 years	4.1
	45–54 years	3.9
	55+ years	3.5

**Table 4:** Correlation between AI confidence level and specialty.

Group	High Confidence in AI	Low/Moderate Confidence	Odds of High Confidence
Specialists (*n* = 90)	72 (80%)	18 (20%)	72/18 = 4.00
General Practitioners (*n* = 60)	27 (45%)	33 (55%)	27/33 = 0.82
Surgeons (*n* = 26)	10 (38%)	16 (62%)	10/16 = 0.63

**Table 5:** Multivariate regression analysis to explore the factors that predict confidence in AI among doctors.

Variable	Beta (β)	Standard Error	t-Value	*p*-Value
Years of Experience	0.05	0.03	1.67	0.097
Age	0.15	0.08	1.9	0.058
Specialty	0.89	0.25	3.56	0.001
Job Displacement Concern	−0.12	0.04	−3	0.003

## Data Availability

Some data are not publicly available due to ethical and privacy restrictions. Other datasets used and/or analyzed during this study are available from the corresponding author upon reasonable request.

## Abbreviations

The following abbreviations are used in this manuscript:

AI	Artificial Intelligence
GP	General Practitioners
OR	Odd Ratios
CI	Confidence Intervals
IRB	Institutional Review Board
KSU	King Saud University
